# SOCIOCULTURAL BARRIERS TO EQUITY IN SOCIAL SUPPORT FOR DEMENTIA CAREGIVING IN MINORITY WOMEN

**DOI:** 10.1093/geroni/igad104.2340

**Published:** 2023-12-21

**Authors:** Heejung Kim, Hyein Kim, Glenna Brewster, Wonshik Chee, Eun-Ok Im

**Affiliations:** Yonsei University, Seoul, Republic of Korea; Yonsei University, Seoul, Republic of Korea; Emory University, Atlanta, Georgia, United States; Emory University, Atlanta, Georgia, United States; Emory University, Atlanta, Georgia, United States

## Abstract

Racial and ethnic minority is growing in dementia caregiving population. This study aimed to identify racial/ethnic differences in social support and sociocultural factors related to caregiving among minority midlife women who were family caregivers of persons living with Alzheimer’s disease (MWPLAD). A secondary data analysis was conducted using the data from a cross-sectional online survey of 172 MWPLADs. Self-report questionnaires were used to collect the data on the caregivers’ sociodemographic and caregiving-related characteristics, social support for caregiving, and sociocultural factors for caregiving. Descriptive statistics, one-way analysis of variance, and hierarchical linear regression were adopted for the data analysis. Non-Hispanic Asian caregivers reported lower social support and greater perceived barrier during dementia caregiving than non-Hispanic African-American and Hispanic caregivers (p ≦ .001). Furthermore, non-Hispanic Asian caregivers reported a higher level of discrimination during caregiving, a lower level of cultural justification for reciprocity, and a lower level of cultural justification for duty than non-Hispanic African-American caregivers (p < .001). After controlling for race/ethnicity and sociodemographic factors, those who received more social support were younger, experienced a lower level of discrimination, and had a higher level of cultural justification for caregiving duties (p < .05). Our study findings support that racial/ethnic differences exist in caregivers’ social support. When enhancing social support among racial/ethnic minority caregivers for dementia, it is necessary to develop specific strategies for encouraging each race/ethnicity group in further large-scale studies and clinical practice in the future.

